# Predictive modeling of molds effective elimination by external inactivation sources

**DOI:** 10.1038/s41598-024-60812-1

**Published:** 2024-05-20

**Authors:** Pavel Demo, Filip Přeučil, Petra Tichá, Mária Domonkos

**Affiliations:** Department of Physics, Faculty of Civil Engineering, Czech Technical University in Prague, Thákurova 7, 166 29 Praha 6, Czech Republic

**Keywords:** Microbiology, Environmental sciences

## Abstract

Presented paper deals with a novel application of the (nonlinear) logistic equation to model an elimination of microscopic filaments types of fungi-molds from affected materials via different external inactivation techniques. It is shown that if the inactivation rate of the external source is greater than the maximum natural growth rate of mycelium, the mold colony becomes destroyed after a finite time. Otherwise, the mycelium may survive the external attack only at a sufficiently large initial concentration of the inoculum. Theoretically determined growth curves are compared with the experimental data for *Aspergillus brasiliensis* mold inactivated by using both cold atmospheric plasma (CAP) and UV-germicidal lamp. Model presented in the article may be applied also to other classes of microorganisms (e.g. bacteria).

## Introduction

The formation and presence of molds on the surfaces (or in the bulks) of various materials (foods, historical artifacts, building materials, etc.^1–5^) represent a serious problem not only from the aesthetic standpoint, but they are potentially responsible for serious human/animal health problems as a consequence of produced mycotoxins (e.g. aflatoxin, collagenase, coagulase, hemolysis, kinases^6–8^). Furthermore, molds may gradually degrade the surface of material during their reproduction cycles resulting in considerable cultural and economical losses. To reduce the proliferation of the molds through the invaded system, various external inactivation techniques are applied in practice. Traditionally used biocidal chemical agents (aldehydes, peroxo and iodine compounds, active chlorine, etc.) exhibit a relatively high index of killing effect in suppression of the molds, but, on the other hand, due to their carcinogenic and toxic effects their application is restricted in more than 30 countries^9^. Among broadly applied methods (in particular, in the food industry) lacking the above disadvantages is heating the damaged system up to sufficiently large temperatures resulting usually in destroying undesirable pathogens in the foods. However, so-called heat-resistant molds may survive this operation^10–12^ to continue spoilage of the heat-processed food. Further increasing of temperature may lead to a decrease of nutritional forces of the sterilized food.

Another innovative treatment of the system damaged by the molds is application of either cold atmospheric plasma (CAP) or the UV-germicidal lamp. While the former one utilizes the synergistic effect of the reactive species produced by CAP (e.g. ozone, singlet oxygen, hydrogen peroxide, hydroxyl radicals, etc.^13,14^) responsible for the lethal effects on the mold and its spores^15^, latter one works with the interaction of radiation of given wavelength with the cells of mold. (It has to be stressed out, however, that the efficacy of UV-lamps is much higher when applied to the bacterial systems).

Laboratory tests of mold inactivation belong rather to long-term experiments (consuming more than 300 h). Consequently, the modeling of this process is welcomed not only due to time-saving reasons, but, in particular, for the possibility to determine straightforwardly the influence of parameters adjustable from the outside on the elimination process.

Regardless of the source of lethal agents (chemical-biocides, fungicides, physical-plasma, UV radiation, heat^10^), in predictive microbiology it is usually assumed that the deactivation process follows first-order kinetics^16–18^. This approach results in exponential decreasing of the number of the microorganisms in the affected sample with time. Or, alternatively, in linear decreasing of survival curves with time^11^. Since one of the important shortcomings of such a linear model is its inability to correctly describe convex or concave survival curves, further models have been developed to cover this gap. In particular, the Weibull distribution and its variations (see, e.g.^17,19^), resp. the Gompertz (sigmoid) model in its alternative forms (e.g.^12,20,21^) belong to those frequently used in microbiology^22^. Novelty of the model presented in this article consists in application of nonlinear logistic equation with the additional term (representing inactivation rate of external source within finite time interval) to describe the extinction of mold mycelium in the sample. This approach also extends number of similar models applied in variety of unrelated fields (e.g. predator–prey interaction with harvesting^23,24^, fishery^25,26^, infectious dynamics^27^ and population models^28^).

## Results

Due to the topologically complex texture of the network-like structure of mycelium on the substrate, the measure of its proliferation has to be defined rather in terms of more continuous characteristics as compared with growth of bacteria. In the presented model, the mold growth is described by the change of its surface coverage with time (e.g.^29–31^).

### Model and calculations

Assume that the surface of substrate in the Petri dish is inoculated with the spores of an initial coverage $\theta (t=0) = \theta _0$. Further proliferation of the inoculate depends on the interplay between the ability of the mold to gradually cover the surface of the substrate and the inactivation rate *I* of the external source. Since the inactivation mechanism is poorly understood (e.g.^20^), it is assumed (within context of the presented phenomenological model) that the mycelium elimination occurs via two-step process:

I. External inactivation source is activated at $t=t_0$, when the part of the substrate is already covered by the growing mycelium and the source is switched off at $t=t_1$.
II. From this moment the mycelium destruction process starts. The damaged colony defends against the external attack through adaptive reparation mechanisms trying to absorb newly established unfavorable environmental conditions. Assume further that this revitalization process terminates at $t=t_2$ and the surviving part of the mycelium (if any) is prepared from this moment for the further proliferation over the substrate. (This adaptation mechanism can be related to the so-called lag-phase of microorganisms evolution^16^ which can span between several hours to several weeks^11^. However, it has to be stressed out that understanding of this complex phenomenon requires deeper microbiological analysis which is out of scope of this article.)

Resulting mathematical picture of this modeled situation in such a way results in (nonlinear) logistic equation

1 $$\begin{aligned} \frac{\textrm{d} \theta (t)}{\textrm{d} t} = r\theta (t)\left[ 1-\frac{\theta (t)}{K}\right] - I\left[ H(t-t_0)-H(t-t_2)\right] \end{aligned}$$

with initial condition

2 $$\begin{aligned} \theta (t=0) = \theta _0. \end{aligned}$$

Above, $\theta (t)$ is the coverage of substrate by mycelium, $H(\cdot )$ stands for the Heaviside generalized function, *r* represents the proportional increase of the surface coverage (due to intrinsic metabolism of mold combined with complex interplay of mold with its environment), *K* is the carrying capacity of the habitat corresponding to the maximum coverage that may be sustained by available resources inside the Petri dish (nutrition, water, living space, etc.) and *I* expresses the inactivation rate of the external source. (Here, the simplest case $I={\text {const.}}$ within interval $\langle t_0, t_2 \rangle$ will be assumed).

This equation can be solved in quadratures or within the context of qualitative analysis allowing to analyze the properties and behavior of the exact solution.

A. $0\le t<t_0$. In this case, the exact solution of the equation
  3 $$\begin{aligned} \frac{\textrm{d} \theta (t)}{\textrm{d} t} = r\theta (t)\left[ 1-\frac{\theta (t)}{K}\right] \end{aligned}$$
  with the known initial condition $\theta (t=0)=\theta _0$ can be expressed as
  4 $$\begin{aligned} \theta (t) = \frac{K\theta _0}{\theta _0 + (K-\theta _0)\textrm{e}^{-rt}}. \end{aligned}$$
B. $t_0\le t<t_2$. The logistic equation within this time interval reads
  5 $$\begin{aligned} \frac{\textrm{d} \theta (t)}{\textrm{d} t} = r\theta (t)\left[ 1-\frac{\theta (t)}{K}\right] - I \end{aligned}$$
  with the initial condition
  6 $$\begin{aligned} \theta (t=t_0) = \frac{K\theta _0}{\theta _0+(K-\theta _0)\textrm{e}^{-rt_0}}. \end{aligned}$$
  Following the qualitative theory of ordinary differential equations (e.g.^32–35^), the first step consists of determination of equilibrium (stationary) points of eq. (5). These points (if exist) correspond to situation when there are no changes in surface coverage by mycelium, i.e., when the following quadratic equation is fulfilled
  7 $$\begin{aligned} \theta ^2 - K\theta + \frac{KI}{r} = 0. \end{aligned}$$
  Since the equilibrium points are real numbers, the condition $I\le rK/4$ has to be satisfied.

When the inactivation rate *I* of the external source is relatively moderate and less than the maximum natural growth rate of mycelium (i.e. $I< rK/4$), then the equilibrium points exist, viz.

8a $$\begin{aligned} \theta _1&= \frac{K}{2}(1-D), \end{aligned}$$

and

8b $$\begin{aligned} \theta _2&= \frac{K}{2}(1+D), \end{aligned}$$

where $D = \sqrt{1-4I/rK}$ and, successively, $\theta _2> \theta _1 > 0$, resp. $\theta _1 < K/2$ and $\theta _2 > K/2$.

If the power of the external inactivation source gradually increases (in limiting case to the critical value $I_{\text {c}} = rK/4$) then only one equilibrium point (double root of eq. (7)) exists:

9 $$\begin{aligned} \theta _3 = \frac{K}{2}. \end{aligned}$$

Finally, for the supercritical values of *I* (i.e., $I > rK/4$) there are no equilibrium points, because the roots of quadratic eq. (7) are complex numbers without any connection to the microbiological reality. In this sense $I_{\text {c}} = rK/4$ represents a threshold value of the external source inactivation rate associated with a dramatic change of the solution of eq. (5).

**Figure 1:**
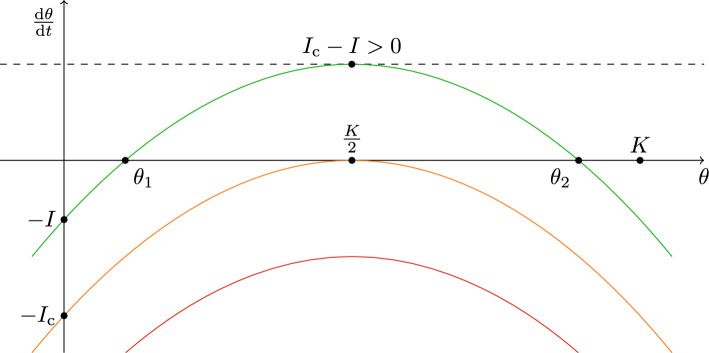
Phase portrait and equilibrium points of eq. (7).

Indeed, as it can be readily deduced from Fig. 1, as *I* increases (i.e., $I_{\text {c}}-I$ decreases) the vertex $[K/2, I_{\text {c}}-I]$ moves downward. As a consequence, the equilibrium points $\theta _1$ and $\theta _2$ become closer together and when the inactivation rate *I* reaches its critical value $I = I_{\text {c}}$, a double root $\theta _3$ appears. Under small external perturbations $\theta _3$ disappears and the solution of the eq. (5) undergoes a change in its behavior: the saddle-node bifurcation (for details, see e.g.^33,36^); $B=I/I_{\text {c}}$ then plays the role of bifurcation parameter.

Having determined the equilibrium points $\theta _1$ and $\theta _2$, the logistic eq. (5) can be expressed in factored form (e.g.^37,38^),

10 $$\begin{aligned} \frac{\textrm{d} \theta (t)}{\textrm{d} t} = \left( -\frac{r}{K}\right) \left[ \theta (t)-\theta _1\right] \left[ \theta (t)-\theta _2\right] \end{aligned}$$

with the exact analytical solution

11 $$\begin{aligned} \theta (t) = \frac{\theta _2(\alpha -\theta _1)-\theta _1(\alpha -\theta _2)\textrm{e}^{-rD(t-t_0)}}{(\alpha -\theta _1)-(\alpha -\theta _2)\textrm{e}^{-rD(t-t_0)}} \end{aligned}$$

satisfying the initial condition

12 $$\begin{aligned} \alpha = \theta (t=t_0) = \frac{K\theta _0}{\theta _0+(K-\theta _0)\textrm{e}^{-rt_0}}, \end{aligned}$$

where $$D = \sqrt{1-B}$$.

Furthermore, as it can be shown with help of Fig. 1 (through identification of regions where $\textrm{d}\theta /\textrm{d}t > 0$, resp. $\textrm{d}\theta /\textrm{d}t < 0$) for subcritical $B < 1$, the equilibrium point $\theta _1$ represents a source, whereas $\theta _2$ is a sink (attractor). It means that $\theta _2$ is an asymptotically stable and $\theta _1$ is an unstable point. Indeed, as it follows from solution (11), when $\alpha > \theta _1$, then $\lim _{t\rightarrow \infty }\theta (t) = \theta _2$ for $r, D > 0$. On the other hand, in the case $\alpha <\theta _1$, then there exists such a time $t=\tau > 0$,

13 $$\begin{aligned} \tau = t_0+\frac{1}{rD}\ln \frac{\alpha -\theta _2}{\alpha -\theta _1} \end{aligned}$$

for which denominator of solution (11) is zero. Thus, $\lim _{t\rightarrow \infty }\theta (t)= -\infty$, since $\theta _2 > \theta _1$ and $\alpha <\theta _1$ (as it is assumed). In practice, it means that if the surface coverage by mycelium at the moment of the external source switch-on is smaller than the unstable equilibrium point, $\theta (t=t_0) = \alpha <\theta _1$, then the mycelium tends to go extinct in finite time. Otherwise, when $\alpha >\theta _1$, the damaged mycelium is able to survive the external source intervention and to continue in the proliferation process after a sufficiently large revitalization period.

C. $t>t_2$. Logistic equation has the form (the external source is in the switched-off regime)

14 $$\begin{aligned} \frac{\textrm{d} \theta (t)}{\textrm{d} t} = r_2\theta (t) \left[ 1-\frac{\theta (t)}{K}\right] , \end{aligned}$$

where $r_2$ is the intrinsic (natural) growth rate of mycelium. (It is assumed, in general, that $r_2$ may differ from *r* due to possible modification of the substrate by the impact of the external source).

Analytical solution of eq. (14) has the form

15 $$\begin{aligned} \theta (t) = \frac{K\beta }{\beta + (K-\beta )\textrm{e}^{-r_2(t-t_2)}}. \end{aligned}$$

satisfying initial condition

16 $$\begin{aligned} \theta (t=t_2) = \beta = \frac{\theta _2(\alpha -\theta _1) -\theta _1(\alpha -\theta _2)\textrm{e}^{-rD(t_2-t_0)}}{(\alpha -\theta _1) -(\alpha -\theta _2)\textrm{e}^{-rD(t_2-t_0)}} \end{aligned}$$

and with $\alpha$ given by (12).

### Comparison with experimental data

It is noteworthy that the model presented in this paper depends, in fact, on six parameters. Four of them (initial surface coverage $\theta _0$, the external source switch on/off times and carrying capacity *K*) are arbitrarily adjustable from the outside. (In our case, *K* can be related to the maximum coverage of substrate inside the Petri dish, i.e., $K = 100\%$). Remaining inputs are uniquely determined by choice of the mold genus (via intrinsic growth rate *r* depending both on metabolism of given mold and its interaction with the environment) and by the parameters of external source (via *I*).

The solution of appropriate logistic equations are tested with a view to validating the model formulation using datasets resulting from experiments with *Aspergillus brasiliensis* inactivated by two different external sources (cold atmospheric plasma (CAP) and UV-germicidal lamp, respectively).

**Figure 2:**
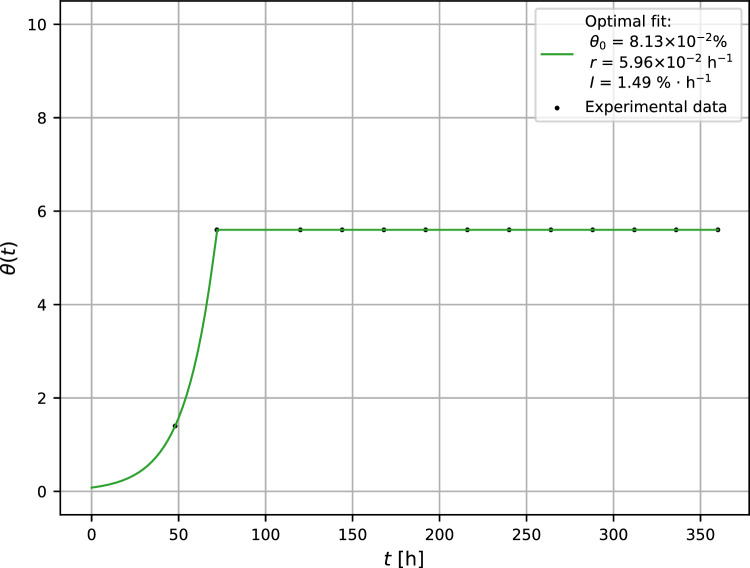
Growth curve of *A. brasiliensis* after plasma intervention at $t_0 = 72$ h after inoculation. The experimental error is less than 4%.

The probability of the mold survival after plasma intervention was studied in two operating modes. In the first experiment, the plasma was activated at $t_0 = 72$ h after initial inoculation of the substrate and with operating time $\Delta t = t_1-t_0 = 10$ min. In this case (as it was proved by a set of numerical experiments), the optimization procedure operates in the regime very sensitive to slight perturbations in the values of optimized fit. Consequently, the optimization of a growth curve under such conditions is a very complex problem and has to be solved via alternative methods. Inserting experimentally obtained points $A = [48\ {\text {h}}, 1.4\%]$ and $B = [72\ {\text {h}}, 5.6\%]$ lying on the exponential part of the growth curve (see Fig. 2) to the eqn. (4), the trivial calculation gives $\theta _0 = 0.0813\%$ and $r = 0.0596\ {\text {h}}^{-1}$.

On the other hand, the growth curve remains unchanged for $t>t_0$ (see Fig. 2) only if $D = \sqrt{1-B} = 0$. Then also $\theta (t>t_0) = \alpha = \theta (t=t_0)$ as it follows from relationships (11) and (12).

Furthermore, for $D = 0$ the bifurcation parameter $B = 1$ and inactivation rate $I = I_{\text {c}} = rK/4 = 1.49\%\cdot {\text {h}}^{-1}$. Consequently, $\theta _1 = K/2 = 50\%$, $\theta (t>t_0) = 5.6\%$. Since $\theta _1> \theta (t>t_0)$, the plasma attack leads to extinction of the mold on the substrate.

**Figure 3:**
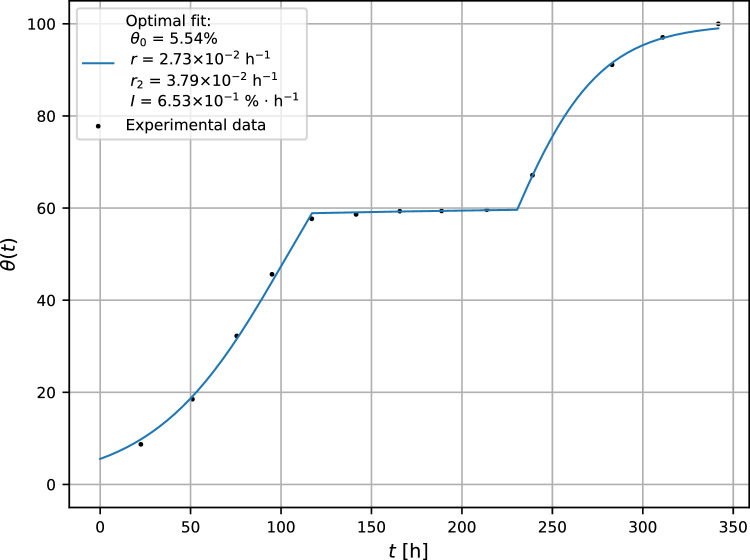
Modeling of the growth curve of *A. brasiliensis*. The experimental error is less than 4%.

Presented model was also applied for a reproduction of *A. brasiliensis* growth when plasma begins to act after $t_0 = 117$ h after MEA inoculation (see Fig. 3). Using both optimal fit values and also relations (8a), resp. (12), one obtains a set of key parameters: critical inactivation rate $I_{\text {c}} = 0.68\%\cdot {\text {h}}^{-1}$, bifurcation parameter $B = 0.96$, unstable equilibrium point $\theta _1 = 39.52\%$ and the surface coverage at $t_0 = 117$ h, $\theta (t = t_0) = 58.89\%$. The inequality $\theta _1 = 39.52\% < 58.89\% = \theta (t = t_0)$ indicates that the mycelium has a clear chance to survive the intervention of lethal agents.

Finally, the mold resistance to extinction caused by other external sources like plasma was studied by the use of a UV-germicidal lamp (operating at the wavelength 253.7 nm where it provides the maximum germicidal effect).

**Figure 4:**
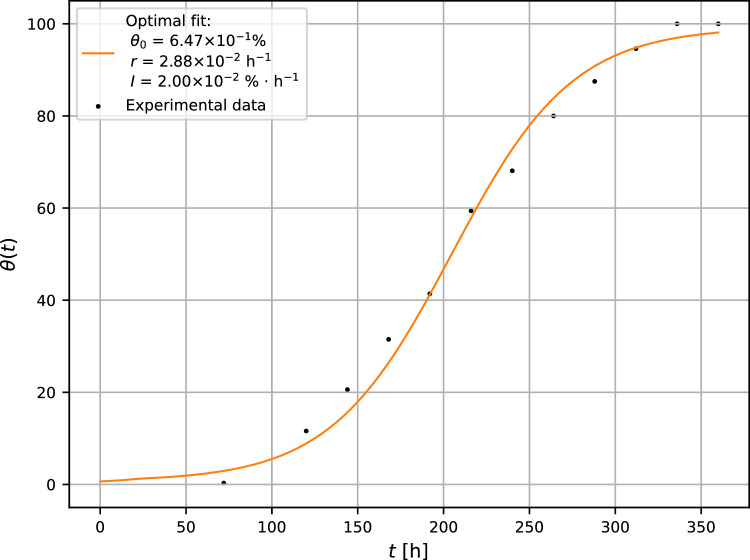
Growth curve of *A. brasiliensis* irradiated by UV-germicidal lamp after 24 h after inoculation. The experimental error is less than 4%.

Partially grown mold was irradiated (in operating time 30 min) by the germicidal UV lamp after 24 h after inoculation. Then the sample was kept in the unchanged environmental conditions up to 360 h and its further development was regularly recorded. The evolution of the irradiated mold is represented in Fig. 4.

For the given values of optimal fit, the following parameters can be determined: critical inactivation rate $I_{\text {c}} = rK/4 = 0.72\%\cdot {\text {h}}^{-1}$, bifurcation parameter $B = 0.0278$ and the unstable equilibrium point $\theta _1 = 0.699\%$ (see expression (8a)). Since the surface coverage $\theta (t_0 = 24\ {\text {h}}) = 1.282\%$ (see relationship (12)) and $\theta _1 = 0.699\%$, then $\theta _1 < \theta (t_0)$, the mycelium exhibits a good chance to survive the external invasion of UV-irradiation and may continue in propagation through the substrate surface.

## Discussion

In many sectors of real life (civil engineering, public health, food-processing industry), the dominant interest is focused on the elimination of the molds proliferation on the surfaces of material applying environmentally acceptable techniques. We herein address, for the first time, a general model of the mold inactivation by use of various external sources exhibiting inactivation rate *I*. (This approach has been applied to specific cases of the inactivation of *A. brasiliensis* using both cold atmospheric plasma and also UV-germicidal lamp). The resulting mathematical picture is a nonlinear logistic balance equation involving inactivation term (operating during a certain time interval) describing the molds growth on the substrate of given carrying capacity. As all calculations are performed by analytical closed form, the presented model is simpler and more effective as compared with other purely numerical approaches. (Although Einstein never said that “Everything should be made as simple as possible, but not simpler,” this statement played a key role in the build-up of the model.)

The principal conclusions drawn from the results presented in this study are as follows:

(A) In the supercritical region $B>1$ (i.e., when the inactivation rate is greater than the critical one), the mycelium always tends to extinction in finite time.
(B) In the subcritical case (when $B<1$) the probability of mycelium survival after plasma intervention depends on the value of its surface coverage $\theta (t_0)$ just at a moment $t=t_0$ of plasma switch-on. When $\theta (t_0)$ is greater than the asymptotically unstable point $\theta _1$, then the mycelium has a good chance to survive the plasma attack. Otherwise, if $\theta (t_0)<\theta _1$, mycelium tends to be destroyed.
(C) As known, the application of UV-germicidal lamps is very effective mostly in eradication of bacteria. In our experiments, the overall ability to terminate the molds proliferation through an invaded system has been studied by using two inactivation external sources (UV-lamp and CAP) operating at different times from inoculation. The treatment with UV irradiation (after 24 h from inoculation) did not stop the growth of mycelium, while the application of CAP even after 72 h after inoculation completely inactivated the mold (in addition, with a shorter exposure time). It has to be stressed out, however, that this complex mechanism of molds inactivation requires a deeper microbiological/biochemical analysis (which is out of scope of presented phenomenologically oriented paper).
(D) Presented model is not necessarily limited only to CAP application for mold inactivation, but also other various sources of lethal agents (heat, UV-radiation, certain chemicals) can be applied.
(E) In this study, the constant inactivation rate *I* was considered in order to simplify appropriate analytical calculations. Consequently, the future extension to overcome this limitation includes the precise modeling of inactivation term related both to real experimental arrangement (e.g. distance of electrode from the mycelium surface, duration of the plasma intervention, power of plasma device, etc.) and also following microbiological characterization of inactivation process.
(F) It has to be pointed out one of the advantages of the presented model. While the real experiments are highly time-consuming (in order of weeks), this approach allows, at least, to obtain related information in order of minutes.

## Methods

**Figure 5:**
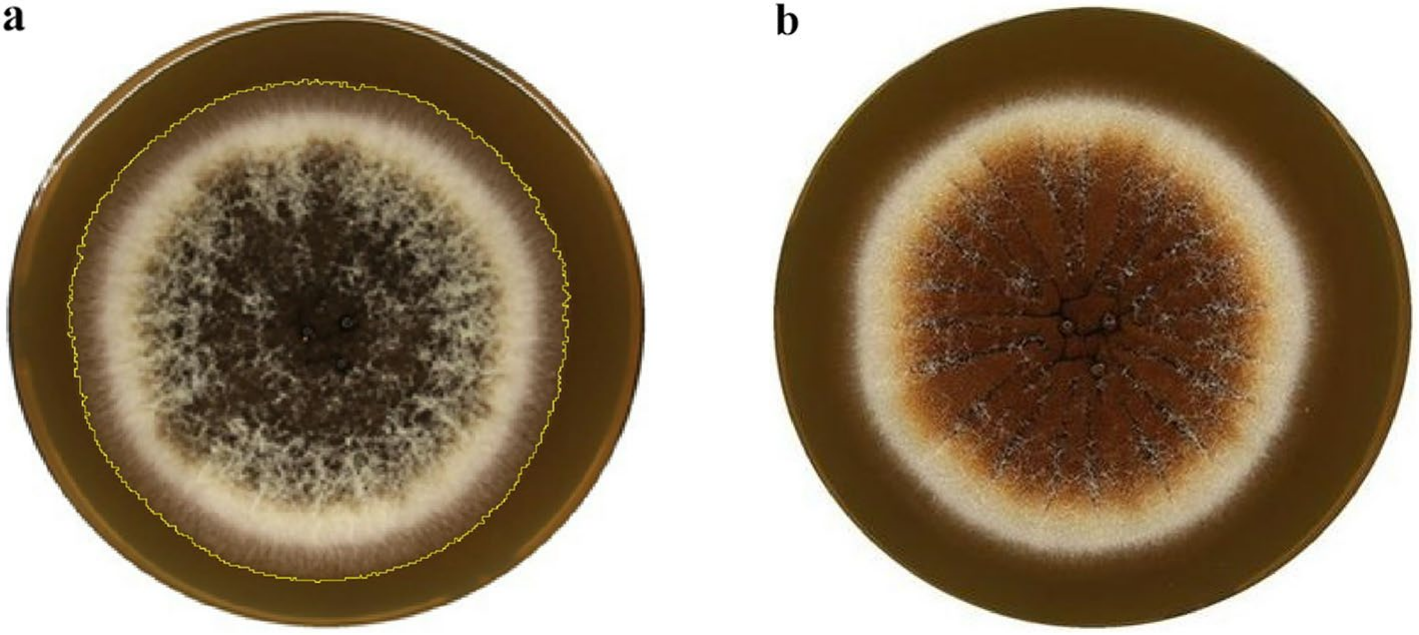
(**a**) Mold detection on MEA (representative reference sample—no plasma treatment). (**b**) Plasma treated *A. brasiliensis* on MEA using diffuse coplanar surface barrier discharge (DCSBD) in ambient air.

In order to compare experimental data with the theoretical results, the mold *Aspergillus brasiliensis CCM8222 (ATCC 16404)* was chosen as a suitable candidate for inactivation procedure (in particular, due to high resolution in coloration between surviving and destroyed parts of mycelium after plasma treatment, see Fig. 5). *Aspergillus brasiliensis* is used as a representative microorganism in accordance with the standard for testing antifungal efficacy as specified in EN 1650. In addition, this species, formerly known as A. niger, is also recommended as a reference microorganism for antifungal susceptibility tests according to EN 1650. However, further research is needed to adapt and validate the model for mixed organisms.

*Aspergillus brasiliensis* suspension was inoculated in known concentration onto the surface of melt extract agar (MEA) using three 0.1 ml droplets. Inoculated plates (Petri dishes with diameter of 90 mm) were placed in an inoculator set to room temperature $23^{\circ }\hbox{C}$. The inoculated plates were then exposed to cold atmospheric plasma (CAP) produced by applying voltage to accelerate electrons, resulting in partial ionization of the surrounding air between the electrode and the surface of mycelium.

The inoculated plates, located 3 mm from the plasma source, were then exposed to cold atmospheric plasma (CAP) produced by applying voltage to accelerate electrons, resulting in partial ionization of the surrounding air between the electrode and the surface of the mycelium. This process leads to the generation of reactive species (charged and neutral particles, photons) that destructively propagate within the mold. The experiments were performed either 72 hours or 117 hours after inoculation using a Diffuse Coplanar Surface Barrier Discharge (DCSBD) device manufactured by Roplass, CZ, with the following process parameters: 300 W of power and a 10-minute treatment time.

The percentage of mold coverage $\theta$ was determined using $\theta = \theta _{\text {m}}/\theta _{\text {A}}\times 100\%$ (where $\theta _{\text {m}}$ is the area covered by mold and $\theta _{\text {A}}$ represents the total area). The evaluation of surface mold growth coverage was performed using image analysis. Samples were withdrawn from the climate chamber on a daily basis to assess mold growth. The surface area was detected by capturing high-resolution photographs of the samples using a camera Canon G1X. Subsequently, the images were analyzed using the Fiji software offering several advantages (simplicity, objective and reproducibility) making it a valuable and widely used tool in research.

Within the comparative test, the mold was also treated by use of germicidal lamp LB 301.2 with operational power $2 \times 30$ W and wavelength 253.7 nm. All samples exposed to UV-C irradiation were treated for 30 min. The distance between the samples and the horizontally oriented UV-C lamp was 30 cm. The intensity of UV irradiation affecting the Petri dishes was approximately $450\,\upmu \hbox{W}/\hbox{cm}^2$.

Curve fitting was done numerically using SciPy python package function scipy.optimize.curve_fit which implements the Levenberg–Marquardt algorithm (LMA) to solve the nonlinear least squares problem. The plots were created using the Matplotlib package.

## Data Availability

The data are available upon request to the authors. For more information, please contact the corresponding author via email at preucil@ipnp.mff.cuni.cz.
